# Mathematical modeling of biohydrogen production via dark fermentation of fruit peel wastes by *Clostridium butyricum* NE95

**DOI:** 10.1186/s12896-024-00925-7

**Published:** 2024-12-18

**Authors:** Norhan Elerakey, Abdel-Hamied M. Rasmey, Akram A. Aboseidah, Heba Hawary

**Affiliations:** Department of Botany and Microbiology, Faculty of Science, Suez University, P.O. Box 43221, Suez, Egypt

**Keywords:** Biohydrogen, *Clostridium butyricum*, Fruit and vegetable peel wastes, Kinetic analysis, Sustainability

## Abstract

**Background:**

Biohydrogen production from agro-industrial wastes through dark fermentation offers several advantages including eco-friendliness, sustainability, and the simplicity of the process. This study aimed to produce biohydrogen from fruit and vegetable peel wastes (FVPWs) by anaerobic fermentative bacteria isolated from domestic wastewater. Kinetic analysis of the produced biohydrogen by five isolates on a glucose medium was analyzed using a modified Gompertz model (MGM). Besides, the feasibility of hydrogen production by *Clostridium butyricum* NE95 using FVPWs as substrates was investigated.

**Results:**

The bacterial isolate NE95 was selected as the highest biohydrogen producer with maximum biohydrogen production (H_max_) of 1617.67 ± 3.84 mL/L, R_max_ (MGM) of 870.77 mL/L/h and lag phase (λ) of 28.37 h. NE95 was phenotypically and genetically identified as *C. butyricum* and its 16 S rRNA gene sequence was deposited in the GenBank under the accession number PP581833. The genetic screening of hydrogenase gene clusters indicated the presence of Fe-Fe hydrogenase gene in *C. butyricum* NE95. *C. butyricum* NE95 showed the ability to produce biohydrogen from different FVPWs, with watermelon and melon peels being the most promising feedstocks for fermentation. It was revealed that using a mixture (1:1, w/w) of watermelon and melon peels as a substrate for *C. butyricum* NE95 significantly increased biohydrogen yield with H_max_ of 991.00 ± 10.54 mL/L, R_max_ of 236.31 mL/L/h, λ of 33.92 h and a high accuracy of R^2^ (0.997).

**Conclusions:**

The study highlights the effectiveness of *C. butyricum* NE95 on the valorization of FVPWs and generates a sustainable source of biohydrogen production.

**Supplementary Information:**

The online version contains supplementary material available at 10.1186/s12896-024-00925-7.

## Background

Depletion of fossil fuels, as well as global warming and greenhouse gases, have prompted the search for non-polluting and renewable energy resources. Hydrogen is regarded as the most efficient eco-friendly energy alternative due to its clean and recyclable nature, lack of carbon emissions during combustion, and high energy density of 122 kJ/g [1]. Compared to traditional non-biological methods of hydrogen production which require a significant amount of energy, biohydrogen production from agro-industrial wastes offers several advantages including lower production cost, appropriate waste disposal, and sustainability [2].

Among agricultural wastes, fruit and vegetable peel waste (FVPW) appears to be an excellent substrate for biohydrogen production via dark fermentation due to its high polysaccharide content (30–70%), low cost, and easy availability throughout the year. Its easy hydrolysis and rapid biodegradation give it superior potential for biohydrogen production compared to other wastes [3]. Inappropriately, landfilling is the most common disposal method around the world for a significant fraction of this waste, leading to landfill leachate and uncontrolled carbon dioxide and methane releases into the atmosphere [4, 5]. Additionally, after decomposing in landfills, FVPW generates approximately 8% of world greenhouse gas emissions [6] Therefore, the utilization of FVPWs as feedstock for dark fermentative biohydrogen production enhances the circular economy.

Microorganisms play a significant role in the biohydrogen production process. Fermentative Bacteria such as *Enterobacter* sp, *Bacillus* sp, and *Clostridium* sp are the most efficient bacteria with higher biohydrogen yields [7]. In practice, members of the *Clostridium* genus are obligate anaerobes that can form spores, which enable them to survive in extreme conditions of temperature, pH and the presence of toxic compounds. As a result, spore-forming *Clostridium* members can be separated from non-spore-forming microbes by pretreating the inoculum. Clostridia produce biohydrogen through the exponential phase of growth, when entering the stationary phase, metabolism shifts towards the formation of metabolite byproducts such as volatile fatty acids (VFA). Compared to facultative anaerobes, clostridia can efficiently utilize a wide range of carbohydrate feedstocks and produce biohydrogen more efficiently [8]. Valdez-Vazquez et al. [9] stated that biohydrogen production is boosted by bioaugmentation with *Clostridium* species. Based on previous publications on biohydrogen production from various substrates using *Clostridium* as fermentative biohydrogen-producing bacteria, *Clostridium butyricum* is the most extensively studied species, accounting for 25% of all publications, followed by C. *beijerinkii*, which accounts for 12% of all publications [10].

Hydrogenase and nitrogenase are two types of metal enzymes involved in the biohydrogen production process through different mechanisms and reactions. hydrogenase can reversibly catalyze the conversion of hydrogen to protons. In contrast, nitrogenase catalyzes the irreversible reaction of nitrogen reduction to ammonia and produces hydrogen as a byproduct in anaerobic environments with low levels of nitrogen [11]. Hydrogenases can be classified into [FeFe] hydrogenases, [NiFe] hydrogenases, and [Fe] hydrogenases based on the different metal ions present in the active center. Hydrogen production rates of [FeFe] hydrogenases are more than 100 times faster than those of [NiFe] hydrogenases, making them logical choices to boost biohydrogen production [12].

The parameters for kinetic analysis of biohydrogen production describe the performance of the fermentation process and vary depending upon the type of microbial inoculum and fermented substrate. It is important to gain a better understanding of how microbial communities ferment carbohydrate-rich substrates, such as organic waste. Each microbial community that produces hydrogen gas has its unique production dynamics, which can help in predicting the biohydrogen production potential of that specific community from a particular substrate. modified Gompertz model (MGM) has been widely used for sustainable biohydrogen production through dark fermentation [13]. The current study aimed to investigate the potential of biohydrogen production using anaerobic bacteria isolated from domestic wastewater via dark fermentation. In addition, biohydrogen production from different fruit and vegetable wastes by the most efficient isolates was evaluated, and the fermentation process was assessed by kinetic analysis using the modified Gompertz model (MGM).

## Materials and methods

### Isolation of anaerobic bacteria

Anaerobic bacteria were isolated from domestic wastewater samples using the dilution pour plate method as described by Abu-elreesh and Abd-el-haleem [14]. Domestic wastewater samples were collected from various sewage plants in the Suez governorate, Egypt. Samples were preheated at 75 °C for 30 min before being diluted. Anaerobic bacterial isolates were cultured on *Clostridium acetobutylicum* (CAB) agar medium, which comprised the following components per liter of distilled water: yeast extract, 4.0 g; tryptone, 1.0 g; K_2_HPO_4_, 1.5 g; asparagine, 0.5 g; resazurin, 1 mL of 0.2% (v/v); MgSO_4_.7H_2_O, 1 g; MnSO4.H_2_O, 0.1 g; FeSO_4_.7H_2_O, 15 mg; NaCl, 0.1 g and glucose 30.0 g [15]. CAB agar medium was supplemented with griseofulvin (50 µg/ml) and nystatin (25 mg/L) to avoid fungal and yeast contamination. The pH of the CAB media was adjusted to 7 before autoclaving using 0.1 N HCl and 0.1 N NaOH. Plates were incubated anaerobically at 37 °C for 48 h. The picked colonies were sub-cultured on fresh agar medium, and preserved at 4 °C.

## Screening bacterial isolates for biohydrogen production (HP)

### Preparation of bacterial inoculum

To prepare bacterial inoculum, one milliliter of a 48-hour-old culture was inoculated into 100 mL of sterilized Reinforced *Clostridium* Medium (RCM) broth which comprised the following components per liter of distilled water: peptone, 10 g; beef extract, 10 g; yeast extract, 3 g; glucose, 5 g; sodium chloride, 5 g; sodium acetate, 3 g; starch, 1 g; and l-cysteine-HCl, 0.5 g [16]. The RCM broths were incubated under anaerobic conditions at 37 °C for 48 h.

## Screening of HP on modified 6% glucose RCM broth

A synthetic glucose medium was utilized to screen the potential of bacterial isolates for HP via dark fermentation. Batch culture was carried out in glass serum bottles (125 mL) with 100 mL as the working volume. Ten milliliters of bacterial inoculum (at a ratio of 10%, v/v) was aseptically added to 90 milliliters of sterilized modified 6% glucose RCM broth. To create anaerobic conditions, glass bottles were flushed with nitrogen, sealed with rubber septa, and incubated at 37 °C for 96 h. The mixture of evolved gas was passed through a 2 M NaOH solution to absorb the maximum amount of carbon dioxide produced. The generated hydrogen gas was measured every 12 h at inverted cylinders using the water displacement method [17]. The collected gas was analyzed by a gas chromatograph (GC) apparatus equipped with a thermal conductivity detector for its composition. A stainless-steel column with a molecular sieve 5 A (80/100 mesh) 2 m x 1/8 inch x 2.1 mm and HayeSepQ (80/100 mesh) 4 m x 1/8 inch x 2.1 mm was used. The column temperature was initially programmed at 50 °C then increased to 200 °C at a rate of 30 °C/min. All gas volumes were measured at 1 atm and 25 °C.

## Identification of the bacterial isolate NE95

### Phenotypic characterization

The colony morphological characteristics including color, shape, consistency, margin, and elevation, were observed on CAB agar plates after 48 h of incubation at 37˚C. Gram staining was conducted to examine bacterial cell shape and other microscopic characteristics [18]. According to Bergey’s Manual of Systematic Bacteriology [19], different biochemical tests, including glucose fermentation, urea hydrolyzation, indole production, Voges–Proskauer test, catalase test, nitrate reduction, H₂S production, citrate utilization, and methyl red tests, were carried out.

## Genotypic identification

### DNA extraction

Quick—DNA Fungal/Bacterial Miniprep Kit (Zymo Research, Irvine, CA, USA) was used for the extraction of genomic DNA according to the method of Abdelrahman et al. [20]. A pure single colony was inoculated into 5 mL of CAB broth medium and incubated at 37 °C under anaerobic conditions for 48 h. After that, the cell pellets were resuspended in the designated volume of Bashing Bead Buffer. Proteinase K was then added, and bacterial cells were incubated at 65 °C for one hour to improve cell lysis. The DNA extraction was performed according to the manufacturer’s protocol for gram-positive bacteria and the extracted DNA was kept at -20 °C for storage.

### 16 S rRNA amplification and taxonomic assignment

Genotypic identification of the bacterial isolate NE95 was done by 16S rRNA gene sequencing. Extracted DNA was used as a template to amplify about 1,400 base pairs of the 16S rRNA gene by PCR using the universal bacterial primers 27F (5’-AGAGTTTGATCCTGGCTCAG) and 1492R (5’- TACGGYTACCTTGTTACGACT) [21]. All PCRs were done with Polymerase Platinum^®^ PCR SuperMix (Thermo Scientific, USA) under the following thermocycling conditions: initial denaturation for 3 min at 95 °C; denaturation for 1 min at 95 °C; annealing at 55 °C for 1 min, and extension at 72 °C for 2 min, followed by a final extension at 72 °C for 10 min.

### Nucleotide sequence analysis

The amplicons were used as a template for bi-directional Sanger sequencing [22]. Sequencing was performed on an ABI Prism 310 Genetic Analyzer (Applied Biosystems). The BLAST (http://www.ncbi.nlm.nih.gov/BLAST/) was used to compare the obtained sequence with other related available sequences in the NCBI database. The sequence was deposited in GenBank and the accession number has been assigned. A phylogenetic tree was conducted using MEGA software (molecular evolutionary genetics analysis [version 11.0]) [23] by the Maximum likelihood method.

### Determination of the growth curve and growth kinetics of *C. Butyricum* NE95

The bacterial isolate *C. butyricum* NE95 was cultivated in a CAB broth medium. A UV-9200 VIS spectrophotometer was used to track optical density changes of the bacterial suspension overtime at 600 nm [24]. The experiment was performed in triplicate. Growth kinetics data was represented by the Monod model as follows [Eq. 1]:

1 $$\:\mu\:=\frac{{\mu}_{{max}}{S}_{i}}{{K}_{s}+{S}_{i}}$$

Where *µ*_max_ is the maximum specific growth rate, *S*_*i*_ is the initial sugar concentration, *K*_s_ is the substrate affinity constant of the Monod model.

### Genetic detection for [Fe-Fe] and [Ni-Fe]-hydrogenase genes in *C. Butyricum* NE95

The bacterial isolate *C. butyricum* NE95 was screened for genes associated with HP. [Fe-Fe] and [Ni-Fe]-hydrogenase genes were amplified by PCR with degenerate primers specific to [FeFe]-hydrogenase (hydA coding sequence with its upstream putative promoter sequence) (UpSt-FW 5′-CATATCAATTCTTTGGCGCT-3′ and RV-1 5′-TTATTTAGTATATTTTAAGTG-3′) [25] and [Ni-Fe]-hydrogenase (HGF133 5′-GTGCGGCGTGAACATTGAAT-3′ and HGR133 5′-TGTTTGCCTTTTTCCAGCGG-3′) based on the published nucleotide sequences of [Ni-Fe]-hydrogenase genes from other *Clostridium* bacterial species, as available in the NCBI database using genomic DNA (50 ng/µL) as a template. All PCRs were conducted using Platinum^®^ PCR SuperMix (Thermo Scientific, USA) (PCR Master Mix (2X) composed of 0.05 U/µL Taq DNA polymerase, reaction buffer, 4 mM MgCl2, 0.4 mM of each dNTP (dATP, dCTP, dGTP, and dTTP), 10 pmol of each primer and DNA (50 ng/µL). PCR conditions were: initial denaturation at 95 °C for 3 min, followed by 35 cycles of denaturation at 95 °C for 1 min, annealing at 55.2 °C for 1 min, and extension at 72 °C for 2 min, with a final extension at 72 °C for 10 min. PCR products were then visualized on 1% agarose gel.

### HP from fruit and vegetable peel wastes (FVPWs) by *C. Butyricum* NE95

Batch fermentative biohydrogen production was conducted using different FVPWs including; banana peels, orange peels, melon peels, watermelon peels, and potato peels. Initially, FVPWs were collected from local fruit shops and markets (Suez governorate, Egypt). The collected FVPWs were chipped and blended in a blender using a suitable amount of sterilized water to obtain the desired concentration of 30% (w/v). Before fermentation, prepared FVPWs were individually preheated in a water bath for 20 min at 50 °C to reduce the natural microbiota in each feedstock. The fermentation process was conducted in glass serum bottles (125 mL) with a working volume of 100 mL. Bacterial inoculum at a ratio of 10%, v/v was added under aseptic conditions into 90 mL of each prepared fruit peel. Serum bottles were incubated under anaerobic conditions at 37 °C for 96 h. Parallel negative controls (with no inoculum but only natural microbiota) and positive controls using a 6% RCM glucose medium were considered. Parallel negative controls underwent the same pretreatment fermentation conditions.

### Enhancing HP by using a mixture of watermelon peel (WMP) and melon peel (MP)

Five mixtures of WMP and MP at different ratios, including (1:1, w/w), (1:2, w/w), (2:1, w/w), (1:3, w/w), (3:1, w/w) were used as substrates for batch biohydrogen production. Batch experiments were conducted using five mixtures, as well as pure peels of watermelon and melon separately. All experiments were preheated at 50 °C, and incubated under anaerobic conditions at 37 °C for 96 h.

### Kinetics study

The Modified Gompertz model (MGM) was applied to the obtained biohydrogen yields. The cumulative biohydrogen (total hydrogen volume) H(t) produced (mL/L) at time t (h) was calculated using [Eq. 2]. The biohydrogen production rate$\:{\:R}_{{H}_{2}}$(mL/L/h) at time t (h) was calculated using [Eq. 3]. The time at which 95% of maximal cumulative biohydrogen was produced (t_95_) was calculated using [Eq. 4] [13].

2 $$H_{(t)}=H_{max}\cdot{\exp}\left\{ {-{\exp}\left[\frac{R_{max}\cdot{e}}{H_{max}}(\lambda-t)\right]} \right\}$$

3 $$\:{R}_{{H}_{2}}={R}_{max}\cdot{\exp}\left\{\left[\frac{{R}_{max}\cdot{e}}{{H}_{max}}\left(\lambda-t\right)+1\right]+\left[\frac{{R}_{max}{\cdot}e}{{H}_{max}}\left(\lambda-t\right)+1\right]+1\right\}$$

4 $$\:{t}_{95}=\frac{{H}_{max}}{{R}_{max}.e}\left[1-\text{ln}(-\text{ln}0.95)\right]+\lambda\:$$

where H_max_ is the maximum cumulative biohydrogen production (mL/L), R_max_ is the maximum biohydrogen production rate (mL/L/h), and e is exp. (1) = 2.71828.

The biohydrogen yield *Y*_*HP/s*_ (mL/g) was calculated based on biohydrogen production concerning the total initial reducing sugar concentration (*S*_*i*_) [Eq. 5]. Volumetric biohydrogen productivity (*Q*_*P*_) (mL/h) was measured based on the actual biohydrogen production in the fermentation time t (h) [Eq. 6] whereas substrate uptake (*Qs*) (g/h) was measured based on consumed reducing sugar in the fermentation time t (h) [Eq. 7].

5 $$\:{Y}_{HP/s}\:(\text{m}\text{L}/\text{g})=dHP/{S}_{i}$$

6 $$\:{Q}_{p}(\text{m}\text{L}/\text{L}/\text{h})=dHP/dt$$

7 $$\:{Q}_{s}(\text{g}/\text{L}/\text{h})=-dS/dt$$

### Analytical methods

The total initial reducing sugars were measured by the dinitrosalicylic acid (DNS) method as described by [26]. Gas chromatography-mass spectrometry (GC-MS) is used to analyze fatty acid esters (FAE) of volatile fatty acids (VFAs) in WMP/MP (1:1, w/w) fermentation media before and after fermentation by *C. butyricum* NE95. Mass spectra were conducted using Shimadzu GCMS-QP2010 (Koyoto, Japan) at an MS ionization voltage of 70 eV, ion source temperature of 220 °C, and equipment current of 60 mA. A 30 m x 0.25 mm i.d. x 0.25 μm film thickness Rtx-5MS fused bonded column equipped with a split-splitless injector was used for GC. At a constant flow rate of 1.37 ml/min, helium gas was used as a carrier gas. Also, the injector temperature was programmed at 280 °C. The column temperature was initially set at 50 °C (isothermal) for 3 min and programmed to 300 °C at a rate of 5 °C/min, then kept constant at 300 °C (isothermal) for 10 min. Diluted samples (1% v/v) were injected with a split ratio of 1: 15. Peak area integration served as the basis for the quantitative measurements.

### Statistical analysis

The data was statistically analyzed using SPSS software version 26.0. All experiments were run out in triplicates. Duncan’s multiple range tests were used to calculate mean values and standard errors. To assess the fitness degree of the acquired data with the studied kinetic model, the determination coefficient factor (R^2^) was employed. Statistical significance was assessed at a 5% level [27]. Tukey’s test was carried out and values were compared at 5% significance level.

## Results

### Screening of isolated bacteria for HP

**Fig. 1:**
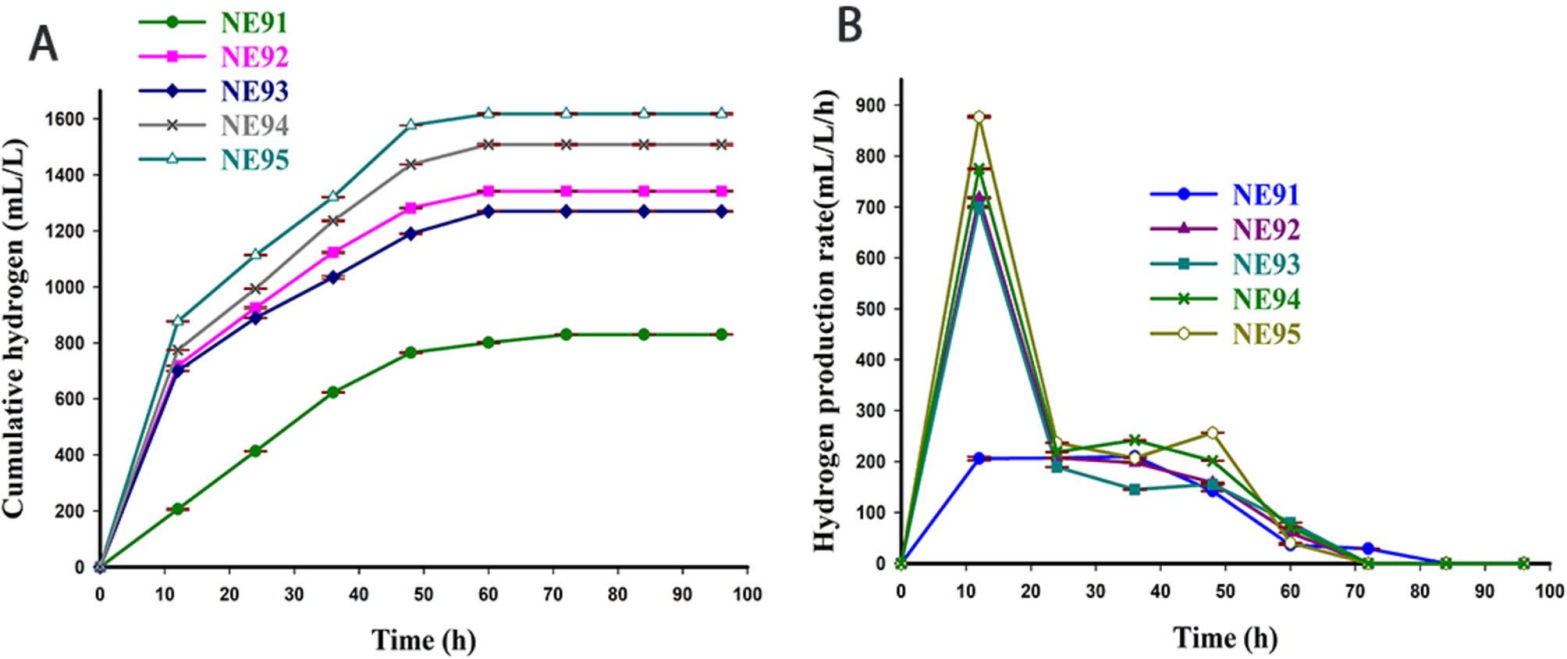
(**A**) HP and (**B**) HPR of bacterial isolates from 6% glucose RCM

Five obligate anaerobic bacterial isolates (NE91, NE92, NE93, NE94 and NE95) were isolated and screened for their potential to produce biohydrogen using 6% glucose RCM broth. A comparative analysis of cumulative (HP) biohydrogen production and biohydrogen production rates (HPR) by five bacterial isolates on 6% glucose RCM over time was shown in Fig. 1. The results graphically showed profound variations in biohydrogen production among bacterial isolates. Bacterial isolate NE95 exhibited the highest HP with a maximum biohydrogen production H_max_ of 1617. 67 ± 3.84 mL/L and experimental maximum biohydrogen production rate R_Emax_ of 877 ± 2.08 mL/L/h while bacterial isolate NE91 showed the lowest HP with H_max_ of 830.00 ± 1.73 ml/L and R_Emax_ of 210 ± 0.70 mL/L/h. NE 95 showed a rapid initial increase of cumulative biohydrogen production during the first 12 h of fermentation reaching approximately 1100 mL/L after 24 h. The final pH of the spent media after fermentation by five bacterial isolates was measured and shown in Table 1. A marked decline in the pH of the fermentation media was recorded to be 5.14, 4.65, 4.67, 4.61, 5.07 for the isolates NE91, NE92, NE93, NE94 and NE95, respectively. Kinetic analysis of HP by different bacterial isolates on a 6% glucose medium using a modified Gompertz model (MGM) revealed that bacterial isolate NE95 was the most efficient biohydrogen producer with the highest R_max_ (MGM) of 870.77 mL/L/h, shortest lag phase (λ) and t_95_ of 28.37, 31.07 h, respectively and high accuracy (R^2^ 0.997). Moreover, the isolate NE95 showed a biohydrogen yield (*Y*_*HP/s*_) of 26.96 mL/g and maximum productivity (*Q*_*P*_) of 26.96 mL/L/h. In contrast, the longest lag phase (32.88 h) and lowest R_max_ (210 mL/L/h) were achieved by bacterial isolate NE 91 with t_95_ of 38.66 h and R^2^ of 0.946 (Table 1). As a result, this bacterial isolate was selected for further identification and characterization studies.

**Table 1:** Kinetic analysis of HP by different bacterial isolates from 6% glucose RCM

Isolate code	Experimental		MGM				Y_HP/s_ (mL/g)	Q_*p*_ (mL/h)	Final pH
	H_max_(mL/L)	*R*_Emax_ (mL/L/h)	λ (h)	*R*_max_ (mL/L/h)	t_95_ (h)	*R* ^2^			
NE 91	830.00^a^ ± 1.73	210^a^ ± 0.70	32.88	210.00	38.66	0.946	13.83	11.53	5.14
NE 92	1342.00^b^ ± 2.91	718^b^ ± 1.86	28.41	714.48	31.14	0.997	22.37	21.65	4.65
NE 93	1269.67^c^ ± 2.60	700^b^ ± 2.08	28.50	692.62	31.15	0.997	21.16	21.87	4.67
NE 94	1508.33^d^ ± 3.71	775^c^ ± 1.20	28.39	774.41	31.23	0.999	25.14	23.20	4.61
NE 95	1617. 67^e^ ± 3.84	877^d^ ± 2.08	28.37	870.77	31.07	0.997	26.96	26.96	5.07

Values are means of three replicates; values followed by the same letters on the same column are not significantly different (*P* < 0.005) in Tukey’s test. *Y*_*HP/s*_ = biohydrogen yield, *Q*_*p*_*=* volumetric biohydrogen productivity

### Identification of the selected bacterial isolate NE95

Phenotypic identification including colony morphology and physiological characteristics of bacterial isolate NE95 were shown in Table 2. It was observed that the isolate exhibited off-white colonies with a circular form, shiny surface, convex elevation, and entire margins possessing semi-transparent mucoid exopolysaccharide capsules. Moreover, NE95 was Gram-positive rods with subterminal and oval spores (S1, supplementary file). Results of biochemical tests indicated that NE95 was negative for catalase, indole, H_2_S, nitrate reduction, oxidase, urease, and methyl red tests. However, the isolate was positive for lactose fermentation, citrate utilization, and Voges-Proskauer tests.

**Table 2:** Phenotypic characteristics of bacterial isolate NE95

Characters	Results
Colony color	Off-white
Colony shape	Circular
Colony appearance	Slimy surface
Colony elevation	Convex
Colony margin	Entire
Colony transparency	Translucent
Cell shape	Spore-forming rod cells
Gram Staining	**+**
Catalase	-
Citrate utilization	**+**
Lactose fermentation	**+**
H_2_S	-
Indole	-
Methyl Red	**-**
Nitrate Reduction	-
Oxidase	-
Urease	-
Voges-Proskauer	**+**

By the comparison of the 16 S rRNA gene sequence of the isolate and others of the GenBank database, the isolate was genetically identified as *Clostridium butyricum* with a similarity of 100% with *Clostridium butyricum* 5368 (MT463464). Our isolate was deposited under the accession number PP581833 in the GeneBank. According to taxonomy, the isolate belonged to the phylum Bacillota, class Clostridia, order Eubacteriales, and family Clostridiaceae. The phylogenetic tree of *C. butyricum* NE95 with other taxonomically and physiologically related species in the GenBank is shown in (S2, supplementary file).

### Determination of the growth curve and growth kinetics of *C. Butyricum* NE95

**Fig. 2:**
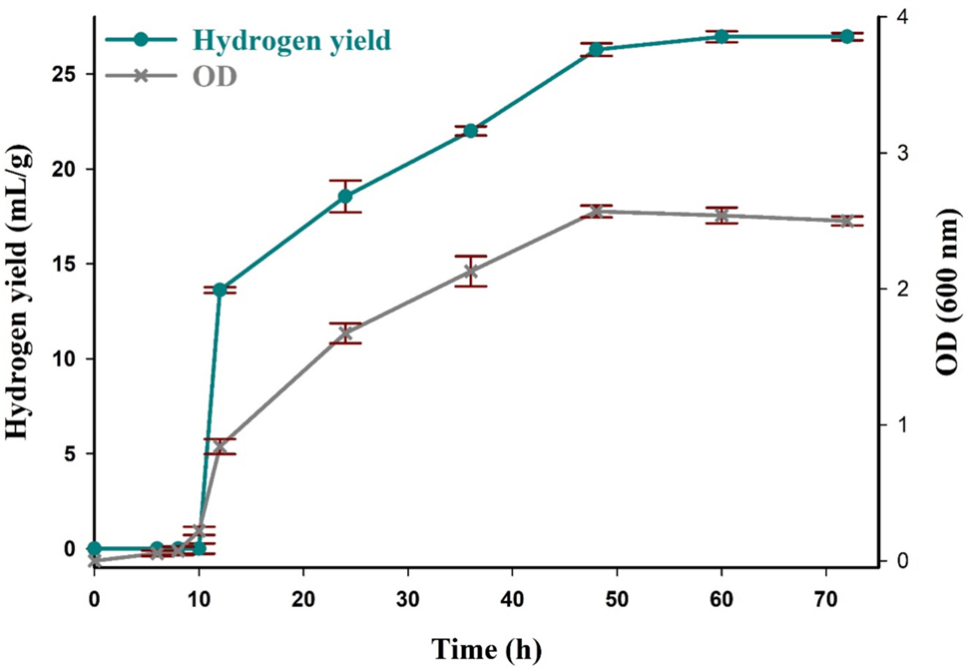
The growth curve and biohydrogen yield of *C. butyricum* NE 95 over time

The profile of cell growth and biohydrogen yield during batch fermentation on CAB medium were shown in Fig. 2. The evolution of hydrogen seemed to begin in the stage of exponential growth (after 10 h), reaching its maximum biohydrogen yield of 26.96 mL/g at 60 h when cell growth had transitioned into the early stationary phase. It was revealed that the maximum specific growth rate (*µ*_max_) was 0.34 h^− 1^ and correlated using the non-linear regression method as follows:

8 $$\:\mu\:=\frac{0.34{S}_{i}}{37.70+{S}_{i}}$$

### Genetic detection for Fe-Fe and Ni-Fe hydrogenase genes in *C. Butyricum* NE95

*C. butyricum* NE95 was screened for [Fe-Fe]-and [Ni-Fe] hydrogenase gene clusters. The isolate showed positive PCR amplification of the [Fe-Fe]-hydrogenase gene and negative for the [Ni-Fe] hydrogenase gene, as shown in (S3, supplementary file). Agarose gel electrophoresis image of PCR amplicons showed a band of expected size (1953 bp DNA fragment) for [Fe-Fe]-hydrogenase gene. The band’s position at the appropriate region in the gel indicates that the [Fe-Fe]- hydrogenase target gene has been detected in *C. butyricum* NE95. The absence of bands in the other lane revealed that the isolate lacks [Ni-Fe]-hydrogenase genes under the same conditions.

### Screening of HP from different FVPWs by *C. Butyricum* NE95

**Fig. 3:**
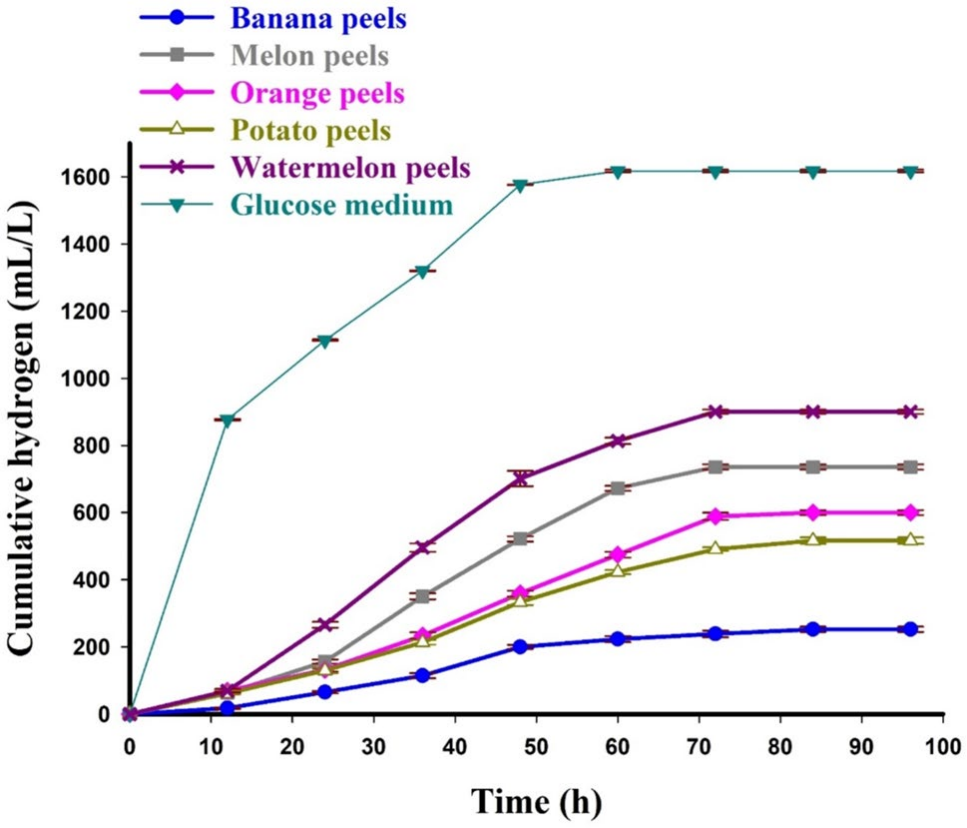
Cumulative biohydrogen production by *C. butyricum* NE95 from different FVPWs along with synthetic glucose medium (positive control)

Cumulative biohydrogen production by *C. butyricum* NE95 from different FVPWs including orange peels, banana peels, melon peels, watermelon peels, and potato peels was demonstrated in Fig. 3. The results showed significant variations in biohydrogen production by *C. butyricum* NE95 from different FVPWs. Biohydrogen production by *C. butyricum* NE95 using watermelon peel (WMP) as a substrate showed the highest H_max_ of 900.67 ± 6.36 mL/L, followed by melon peel (MP) with H_max_ of 736.33 ± 8.25 mL/L. The lowest H_max_ (252.33 ± 7.88 mL/L) was observed when using banana peel as a substrate. Furthermore, the biohydrogen production was much lower for all wastes in cases with no inoculum (negative control). kinetic analysis using MGM of HP by *C. butyricum* NE95 from FVPWs was demonstrated in Table 3. Kinetic parameters indicated that WMP was the best substrate for HP by *C. butyricum* NE95 with the highest R_max_ of 227.63 mL/L/h and shortest lag phase and t_95_ of 33.97, 39.69 h, respectively. MP was the second effective substrate for HP by *C. butyricum* NE95 with R_max_ of 193.89 mL/L/h, λ of 34.21 h, t_95_ of 39.74 h and R^2^ of 0.991. In contrast, the longest λ (40.23 h) and the longest R_max_ (84.91 mL/L/h) were obtained from HP from banana peels with t_95_ of 44.53 h and R^2^ of 0.953.

**Table 3:** Kinetic analysis of HP by *C. Butyricum* NE95 from different FVPWs using modified Gompertz model (MGM)

Substrate	Inoculum	Experimental		MGM			
		H_max_(mL/L)	*R*_Emax_ (mL/L/h)	λ (h)	*R*_max_ (mL/L/h)	t_95_ (h)	*R* ^2^
Control	NE 95	1617.67^a^ ± 6.66	877^a^.00 ± 2.08	28.37	870.77	31.07	0.997
Banana peels	NC	70.33^b^ ± 1.45	27.67^b^ ± 2.03	27.93	26.41	31.64	0.916
	NE 95	252.33^c^ ± 7.88	85.67^c^ ± 2.62	40.23	84.91	44.53	0.953
Melon peels	NC	76.33^d^ ± 2.33	25.00^d^ ± 0.82	28.33	24.20	32.79	0.964
	NE 95	736.33^e^ ± 8.25	194.33^e^ ± 5.10	34.21	193.89	39.74	0.991
Orange peels	NC	94.00^f^ ± 2.31	34.33^f^ ± 1.48	22.32	31.57	26.32	0.974
	NE 95	600.00^g^ ± 7.51	125.67^g^ ± 2.91	39.23	120.81	46.20	0.988
Potato peels	NC	39.33^h^ ± 1.76	12.33^h^ ± 0.84	22.12	12.21	26.78	0.998
	NE 95	516.33^i^ ± 9.24	120.00^g^ ± 3.17	39.56	116.38	45.84	0.994
Watermelon peels	NC	59.33^j^ ± 2.19	21.33^i^ ± 1.86	22.11	19.65	26.17	0.977
	NE 95	900.67^k^ ± 6.36	230.00^j^ ± 5.80	33.97	227.63	39.69	0.987

Control = Synthetic glucose medium (6% glucose RCM), NC = Negative Control (without inoculum), MGM = modified Gompertz model, values are means of three replicates; values followed by the same letters on the same column are not significantly different (*P* < 0.005) in Tukey’s test

### Enhancing HP by using a mixture of WMP and MP

**Fig. 4:**
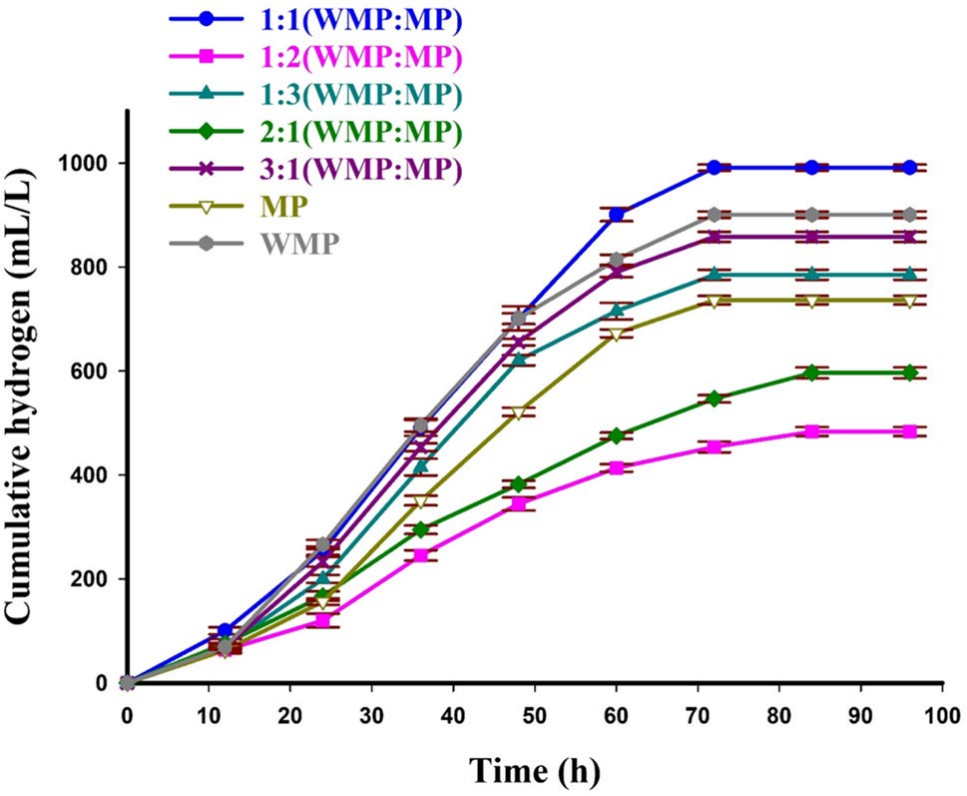
Cumulative biohydrogen production from different mixtures of WMP and MP by *C. butyricum* NE95

The two highly efficient substrates (WMP and MP) for HP by *C. butyricum* NE95 were mixed at various ratios to enhance biohydrogen yield. Figure 4 showed cumulative HP from different mixtures of WMP and MP using *C. butyricum* NE95 over time. Experimental results showed that the highest HP was achieved using a mixture (1:1, w/w) of WMP and MP with H_max_ of 991.00 ± 6.08 mL/L and R_Emax_ of 236.33 ± 7.39 mL/L/h. Kinetic analysis of HP by this mixture showed the highest R_max_ of 236.31 mL/L/h, shortest λ (33.92 h), and t_95_ of 40.05 h with high model fit (R² = 0.997) (Table 4). Remarkably, *C. butyricum* NE95 produced high biohydrogen yields using a mixture of WMP: MP (3:1, w/w) respectively as a substrate with H_max_ of 858 ± 9.71 mL/L, R_Emax_ of 220.33 ± 3.51 mL/L/h, R_max_ of 219.62 mL/L/h, **λ** of 33.99 h, t_95_ of 39.68 h and R^2^ 0.998. In contrast, the lowest R_max_ (125.31mL/L/h) and the longest lag phase (39.68 h) were obtained using a mixture of WMP: MP (1:2. w/w) as a substrate, respectively. It was revealed that the maximum biohydrogen yield (*Y*_*HP/s*_) (69.11 mL/g) and maximum *Qs* (0.178 g/L/h) were achieved by *C. butyricum* NE95 using a mixture of WMP and MP (1:1, w/w) while the lowest *Y*_*HP/s*_ (34.01 mL/g) and *Qs* (0.119 g/L/h) were obtained using a mixture of WMP and MP (1:2, w/w).

**Table 4:** Kinetic analysis of HP by *C. Butyricum* NE95 from different mixtures of WMP to MP

Mixture	Experimental		MGM				*S*_*i*_ (g/L)	*Qs* (g/L/h)	*Y*_*HP/s*_ (mL/g)
	H_max_ (mL/L)	*R*_Emax_ (mL/L/h)	λ (h)	*R*_max_ (mL/L/h)	t_95_ (h)	*R* ^2^			
Watermelon peels (WMP)	900.67^a^ ± 6.36	230.00^a^ ± 5.80	33.97	227.63	39.69	0.987	14.7 ± 0.27	0.172	61.27
Melon peels (MP)	736.33^b^ ± 8.25	194.33^b^ ± 5.10	34.21	193.89	39.74	0.991	13.98 ± 0.15	0.156	52.67
1:1 (WMP: MP)	991.00^c^ ± 6.08	236.33^a^ ± 7.39	33.92	236.31	40.05	0.997	14.34 ± 0.28	0.178	69.11
1:2 (WMP: MP)	483.67^d^ ± 8.57	125.33^c^ ± 5.16	39.68	125.31	45.32	0.977	14.22 ± 0.10	0.119	34.01
1:3 (WMP: MP)	785.33^e^ ± 9.64	215.33^d^ ± 8.33	34.11	214.29	39.44	0.985	14.16 ± 0.35	0.162	60.59
2:1 (WMP: MP)	596.67^f^ ± 10.53	127.67^c^ ± 3.51	39.38	127.65	46.21	0.994	14.45 ± 0.18	0.129	41.29
3:1 (WMP: MP)	858.00^a^ ± 9.71	220.33^ad^ ± 3.51	33.99	219.62	39.68	0.998	14.52 ± 0.25	0.167	59.09

MGM = modified Gompertz model, values are means of three replicates; values followed by the same letters on the same column are not significantly different (*P* < 0.005) in Tukey’s test. *Y*_*HP/s*_ = hydrogen yield, *S*_*i*_*=* initial reducing sugar concentration,

*Qs* = substrate uptake

### Gas chromatography-mass spectrometry (GC-MS) analysis of WMP/MP medium extract mixture (1:1, w/w) before and after fermentation by *C. Butyricum* NE95

The results of GC-MS analysis of WMP/MP (1:1, w/w) medium extract before (control) and after fermentation by *C. butyricum* NE95 were shown in Table 5. It was revealed that the control medium mainly contains fatty acids contains fatty acids such as linoleic acid (Grape seed oil) (10.82%), palmitic acid ethyl ester and palmitic acid (13.89), stearic acid ethyl ester (4.09%), Bis(2-ethylhexyl) phthalate (20.62%) (S4, supplementary file). After the fermentation process, certain organic acids such as eicosyl trifluoroacetate (3.37%), citric acid, triethyl ester (1.04%), hexacosyl heptafluorobutyrate (2.02%), tetratriacontyl heptafluorobutyrate (2.22%) were detected (S5, supplementary file). Palmitic acid ethyl ester and palmitic acid levels were decreased and detected at a percentage of 13.44%.

**Table 5:** Gas chromatography-mass spectrometry (GC-MS) analysis of WMP/MP medium extract mixture (1:1, w/w) before and after fermentation by *C. Butyricum* NE95

Medium	Compound	Formula	Retention time	Area	Area (%)
Mixture of WMP and MP control medium	Bis(2-ethylhexyl) phthalate	C_24_H_38_O_4_	44.418	14,282,180	20.62
	9,12-Octadecadienoic acid (Z, Z) (Grape seed oil) (linoleic acid)	C_18_H_32_O_2_	37.765	7,491,950	10.82
	Hexadecanoic acid, ethyl ester (palmitic acid, ethyl easter)	C_18_H_36_O_2_	34.536	6,242,070	9.01
	Linolenic acid, ethyl ester	C_20_H_34_O_2_	37.902	5,789,825	8.36
	Palmitic acid	C_16_H_32_O_2_	33.992	3,381,830	4.88
	Stearic acid, ethyl ester	C_20_H_40_O_2_	38.323	2,831,474	4.09
	Pentacontanoic acid, ethyl ester	C_52_H_104_O_2_	48.039	2,214,423	3.20
Mixture of WMP and MP spent medium	Bis(2-ethylhexyl) phthalate	C_24_H_38_O_4_	44.426	24,145,631	23.14
	Hexadecanoic acid, ethyl ester (palmitic acid, ethyl easter))	C_18_H_36_O_2_	34.537	10,104,246	9.68
	Stearic acid, ethyl ester	C_20_H_40_O_2_	38.326	7,010,313	6.72
	Linolenic acid, ethyl ester	C_20_H_34_O_2_	37.886	6,293,512	9.87
	Palmitic acid	C_16_H_32_O_2_	33.990	3,926,689	3.76
	Eicosyl trifluoroacetate	C_22_H_41_F_3_O_2_	47.997	3,515,146	3.37
	Hexacosene	C_26_H_52_	45.004	3,137,158	3.01
	Citric acid, triethyl ester	C_12_H_20_O_7_	27.468	1,088,892	1.04
	Hexacosyl heptafluorobutyrate	C_30_H_53_F_7_O_2_	50.785	2,108,297	2.02
	Tetratriacontyl heptafluorobutyrate	C_38_H_69_F_7_O_2_	53.409	1,434,813	2.22

## Discussion

In the present study, five obligate anaerobic bacterial isolates were isolated from sewage plants. The isolates could produce biohydrogen with significant variations in biohydrogen concentration using RCM glucose medium. Obligate anaerobes are known for their high biohydrogen-producing performance, making them excellent microorganisms for biohydrogen production. The most commonly used obligate anaerobes for dark fermentative biohydrogen production are species of *Clostridium* and *Thermoanaerobacterium* [28]. The bacterial isolate designated as NE95 outperformed all other isolates in terms of cumulative H_2_ production and hydrogen production rate. This isolate was identified as *Clostridium butyricum* based on genetic analysis using 16 S rRNA gene sequencing, along with morphological observation and biochemical tests. The phenotypic characteristics of NE95 in our study were matched with the results of *C. butyricum* characterization of previous studies by Lan et al. [29] and Wang et al. [30] as a sporulating Gram-positive bacillus which is negative for nitrate reduction, hydrogen sulfide and positive for glucose fermentation. NE95 was consistent with the physiological characteristics of *C. butyricum* described in Bergey’s Manual.

The 16 S rRNA gene sequence of *C. butyricum* strain NE95 has been deposited in GenBank under accession number PP581833 and was genetically clustered with other strains of *C. butyricum*, which supports its identity. Phylogenetic analysis highlights the identification of NE95 as *C. butyricum* and the close evolutionary relationship between this isolate and other clostridial species. This branching tree indicates a common ancestor of *C. butyricum* NE95 and others of *Clostridium* include *C. acetobutylicum* and *C. saccharobutylicum*. This classification is consistent with previous studies by Lan et al. [29]. The results indicate that *C. butyricum* NE95 is a promising biohydrogen producer, which is consistent with previous studies on the efficiency of *Clostridium butyricum* in producing biohydrogen via dark fermentation [8, 31]. The relationship between biohydrogen yield and the growth profile of *C. butyricum* NE 95 was studied. Biohydrogen production started in the exponential growth of *C. butyricum* NE 95 reaching its maximum yield when cell growth had transitioned into the early stationary phase. This demonstrated that biohydrogen production during the assimilation of carbon substrate for biomass synthesis was not a preferred event [32].

*C. butyricum* NE95 was genetically screened for the presence of [Fe-Fe] and [Ni-Fe]-hydrogenase gene clusters. The isolate was positive for [Fe-Fe]-hydrogenase gene by PCR amplification. This indicates that *C*. *butyricum* NE95 produces biohydrogen through the enzymatic activity of Fe-Fe hydrogenase genes which catalyze H_2_ formation by the reduction of protons. This is consistent with Tolvanen et al. [33] who revealed that HP in C*lostridium* is associated with the activity of [FeFe]-hydrogenase genes. [FeFe]-hydrogenases are frequently found in anaerobic bacteria and well-studied in *Clostridium* species [34, 35]. *Clostridium* is regarded as the primary biohydrogen producer among biohydrogen-producing bacteria due to its hydA enzymes. [FeFe]-hydrogenases (HydA) are associated with the synthesis of H_2_ and serve as the most effective biocatalyst for biohydrogen production with a turnover frequency of up to 10^4^/s [36, 37]. Our isolate was [Ni-Fe] hydrogenase negative by PCR. This may be due to the limited presence of this enzyme in *Clostridium* representatives and its prevalence in archaea and other types of bacteria [38].

*Clostridium* species produce biohydrogen from reducing powers by anaerobic fermentation using hydrogenase. They generate hydrogen through the pyruvate: ferredoxin oxidoreductase (PFOR) pathway [39]. *Clostridium* species’ [Fe-Fe]-hydrogenases have ferredoxin-like domains that are thought to play a significant role in enhancing interaction with ferredoxins and efficiently transferring electrons to the hydrogenase active site [40].

Our study has explored a different way to produce biohydrogen by using different agricultural wastes, specifically FVPWs such as watermelon, melon, orange, banana, and potato peels, as alternative substrates. This aligns with Dwivedi et al. [41], who utilized fruit and vegetable waste as substrates for dark fermentative biohydrogen production, replacing polysaccharide substrates. This offers an inexpensive and sustainable substrate for biohydrogen production, while also positively contributing to waste management in an eco-friendly manner. The use of fruit and vegetable waste as an adequate feedstock for clostridial fermentation due to its high carbon and volatile solid content which could be metabolized by fermentative bacteria [6]. Abubakar et al. [42] pointed out that fruit and vegetable wastes are ideal feedstock for biohydrogen production due to their biodegradability, carbohydrate-rich, and moisture content. Several studies investigated using FVPWs such as banana, orange, melon, and potato peels for HP [43–46]. Therefore, using such sustainable substrates makes the fermentation process more economical and reduces the overall cost of biohydrogen production. Our findings showed that using watermelon peels (WMP) as a substrate for clostridial fermentation demonstrated superior performance with the highest cumulative biohydrogen production volume and rate, revealing that it’s a highly efficient and promising substrate for *C. butyricum* NE95 among the tested substrates. WMP contains high carbohydrate content (40%) and moisture content (18.4%) [47]. MP was the second effective substrate for *C. butyricum* NE95 according to our findings. Melon waste has high sugar content (30.42%), hemicellulose (22.71%), cellulose (19.01%), lignin (8.26%), and soluble starch (17.22%) [44]. The obtained data showed that HP using a mixture of WMP and MP (1:1, w/w) as a substrate for *C*. *butyricum* NE95 was significantly higher concerning pure substrates. Kinetic analysis of HP by this mixture showed the highest R_max_ and the shortest λ. This indicates a synergistic effect, improving biohydrogen production more than pure substrates used separately. Using this mixture as a substrate for HP by *C*. *butyricum* NE95 improves HP by 10.03% compared to using sole WMP and by 34.59% compared to using sole MP. This may be because it provides more fermentable sugars and nutrients that favor fermentative bacterial metabolism, resulting in high biohydrogen yields. Turhal et al. [48] revealed that a mixture of melon and watermelon can enhance biohydrogen production. The mixture of WMP/MP in a 1:1 weight ratio serves as an effective substrate for *C. butyricum* NE95. However, the isolate achieved greater biohydrogen yield using glucose as the sole substrate compared to the fruit peel mixture. The reason might be the complex structure of FVPWs which makes the biodegradable sugars less accessible resulting in a lower biohydrogen production. Therefore, pretreatment is required to maximize the production of fermentable sugars that can be easily absorbed by biohydrogen-producing microbes and biologically converted into biohydrogen [49, 50].

Fatty acids like palmitic acid, linoleic acid, stearic acid, and linolenic acid were recorded through GC-MS analysis of the mixture of WMP and MP control medium, which was consistent with the previous studies of Petchsomrit et al. [51] and Silva et al. [52]. Certain organic acids, such as acetic acid, butyric acid, and citric acid, were recorded after fermentation. This indicated that biohydrogen is produced by *C. butyricum* NE95 when butyric acid and acetic acid are produced [53]. Also, the presence of some long-chain fatty acids such as linoleic acid and palmitic acid in WMP and MP might enhance the biohydrogen yields by inhibiting hydrogen-consuming microbes and redirecting electron equivalent to biohydrogen production [54–56]. Saady et al. [57] demonstrated that palmitic acid establishes conditions favoring the acetate pathway, through which maximum biohydrogen yield was observed. This revealed the efficient use of WMP and MP peels as a substrate for *C. butyricum* NE95 to produce biohydrogen and develop a sustainable renewable energy source.

## Conclusions

The present study emphasizes the effectiveness of *Clostridium butyricum* NE95 in biohydrogen production from fruit and vegetable peel wastes (FVPWs). Watermelon and melon peels were found to be the most favorable substrates for dark fermentative biohydrogen production by *C. butyricum* NE95. Our results demonstrated that using a mixture (1:1, w/w) of watermelon and melon peels as a feedstock for *C. butyricum* NE95 effectively boosts biohydrogen production. The study highlights the utilization of FVPWs in biohydrogen production to achieve both waste management and a sustainable energy source.

## Electronic supplementary material

Below is the link to the electronic supplementary material.

Supplementary Material 1

## Data Availability

16 S rRNA nucleotide sequence of the bacterial isolate C. butyricum NE95 was deposited in the GenBank database and assigned the accession number PP581833. All the analyzed and generated data are included in this study.

## Abbreviations

FVPWs: Fruit and vegetable peel wastes
MGM: Modified Gompertz model
H_max_: Maximum hydrogen production
R_max_: Maximum hydrogen production rate
λ: Lag phase
R_Emax_: Experimental maximum hydrogen production rate
HP: Hydrogen production
HPR: Hydrogen production rate
WMP: Watermelon peel
MP: Melon peel
FAE: Fatty acid esters of
VFAs: Volatile fatty acids
Q_P_: Volumetric hydrogen productivity
S_i_: Initial reducing sugar concentration
Y_HP/s_: Hydrogen yield
µ_max_: Maximum specific growth rate
K_s_: The substrate affinity constant of the Monod model
GC-MS: Gas chromatography-mass spectrometry
