# Supplementary material for: Mathematical modeling of biohydrogen production via dark fermentation of fruit peel wastes by Clostridium butyricum NE95
Source: BMC Biotechnol. 2024 Dec 18;24:105. doi: 10.1186/s12896-024-00925-7 (PMC11653998; doi:10.1186/s12896-024-00925-7)
Supplement: Supplementary file 1 — Supplementary Material 1 [file 12896_2024_925_MOESM1_ESM.docx]

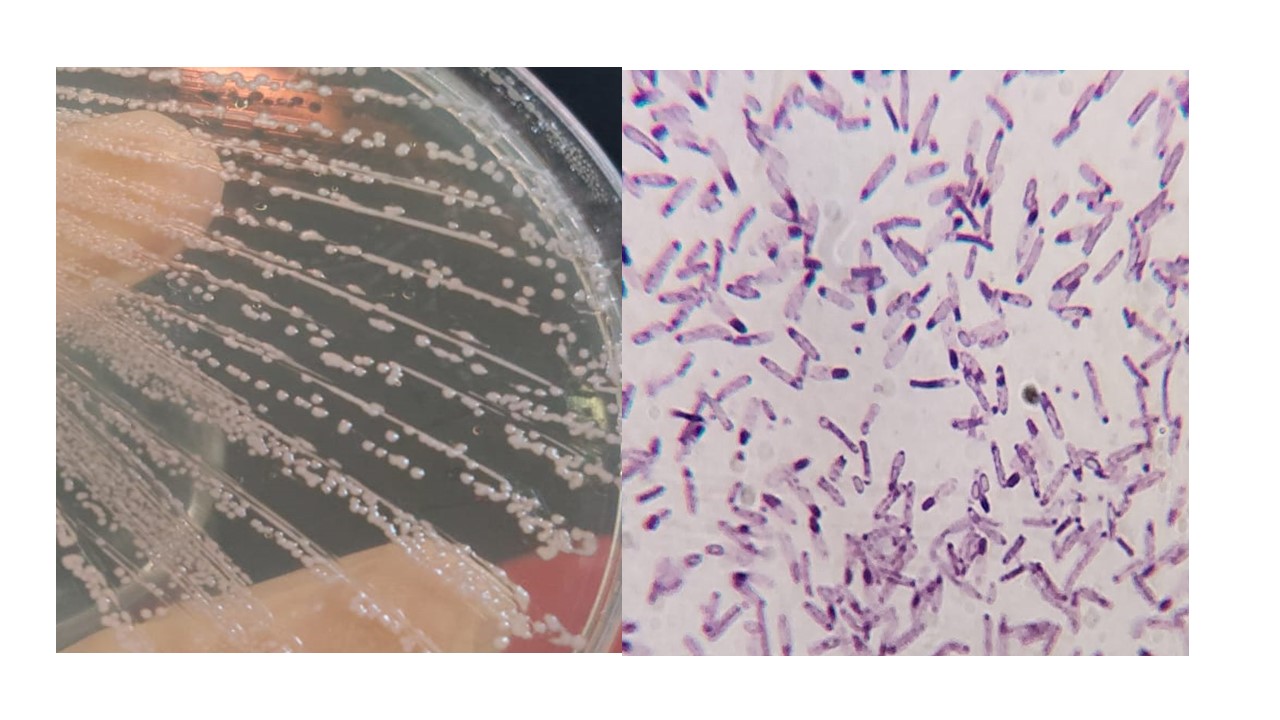


**Fig. S1.** Morphological characteristics of bacterial isolate NE95

**
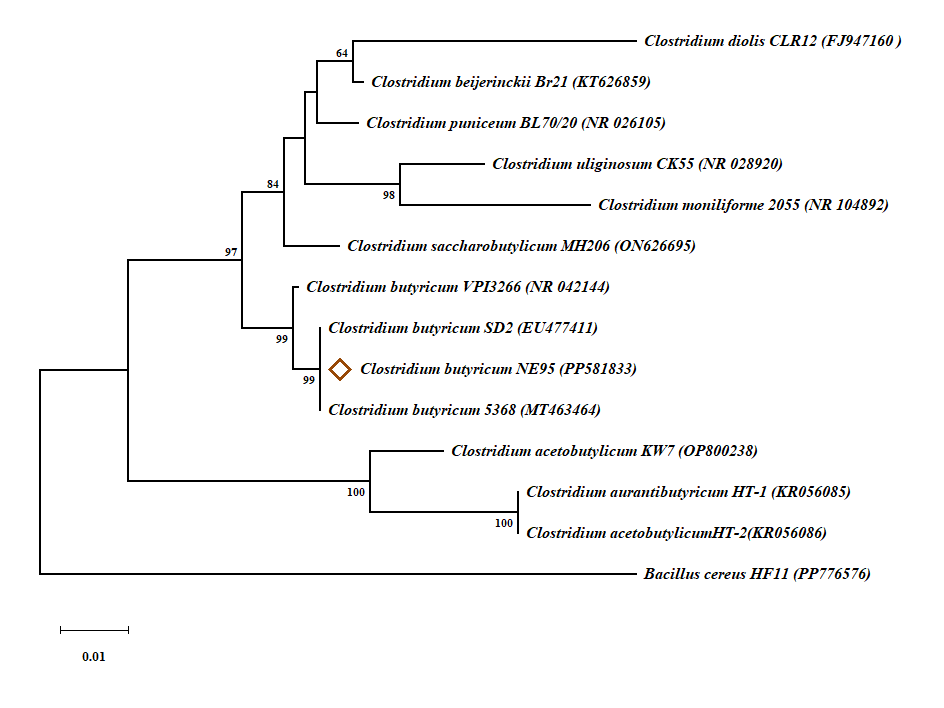
**

**Fig. S2.** Molecular rooted Phylogenetic 16S rRNA gene tree analysis of *Clostridium* species by Maximum Likelihood method. The tree was rooted using the 16S rRNA partial gene sequence of *Bacillus cereus* HF11 (PP776576).


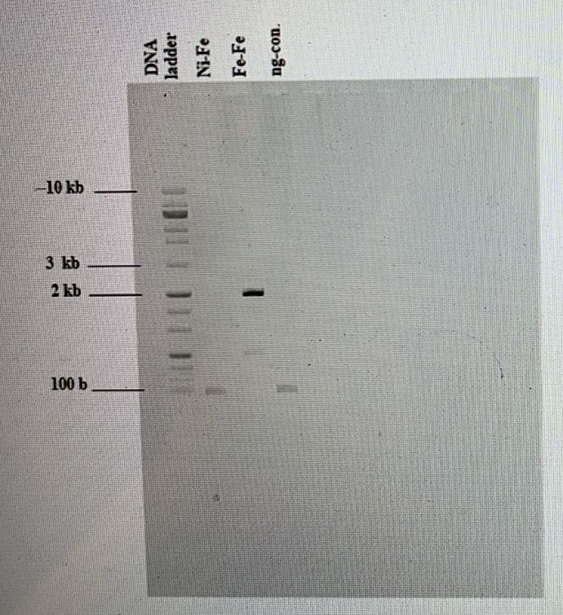


**Fig. S3.** PCR amplicons of [Fe-Fe]-and [Ni-Fe] primers for *C. butyricum* NE95


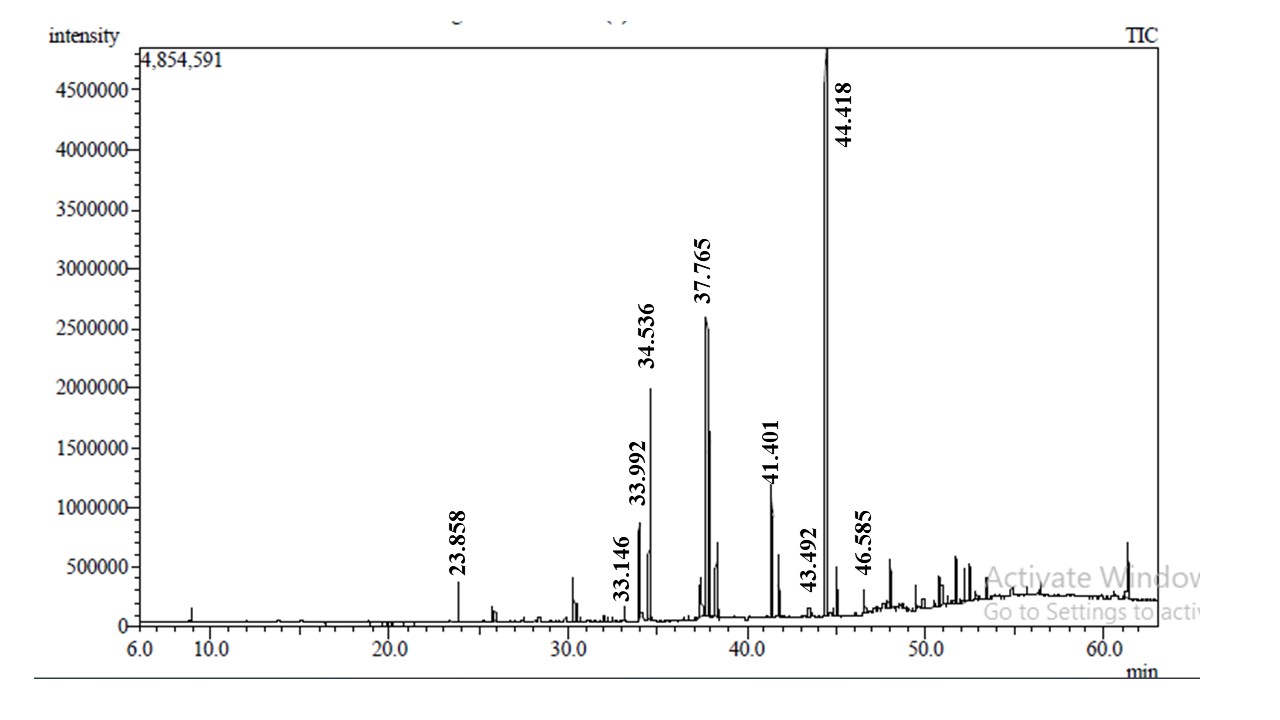


**Fig. S4.** GC-MS analysis of mixture of WMP and MP (1:1, w/w) control medium before fermentation


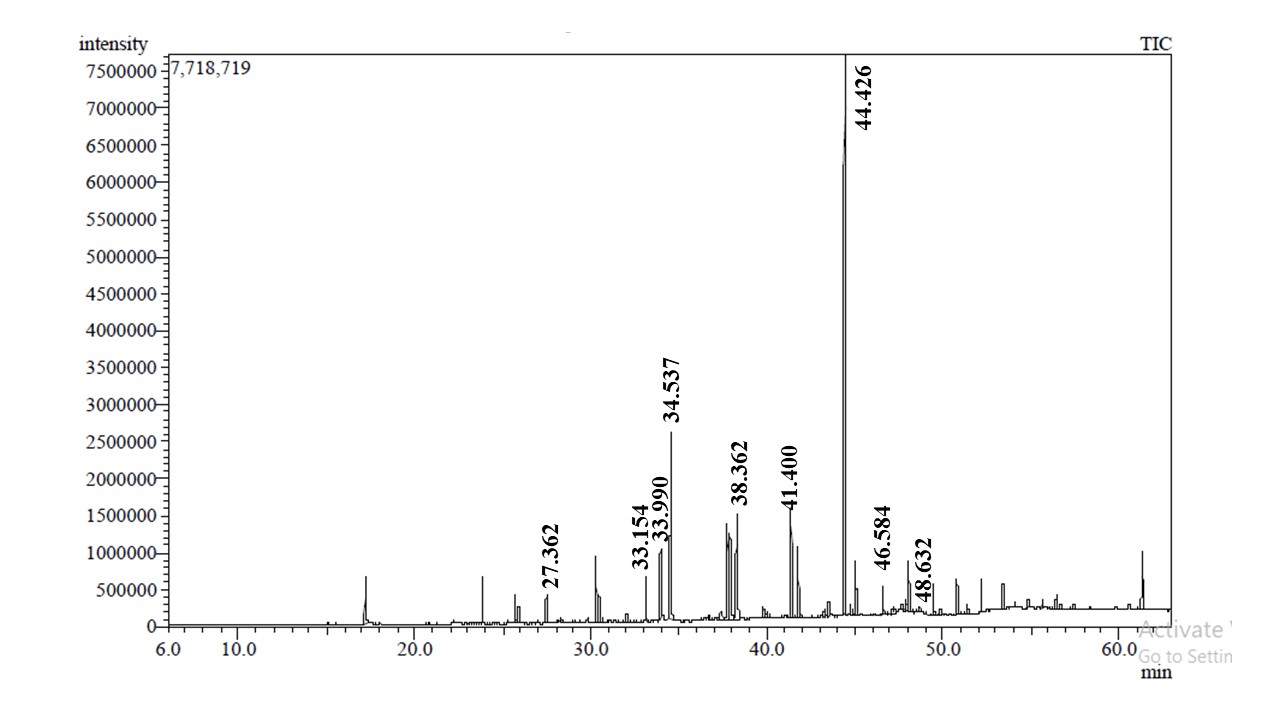


**Fig. S5.** GC-MS analysis of mixture of WMP and MP (1:1, w/w) spent medium after fermentation
